# Tanshinone I inhibits tumor angiogenesis by reducing STAT3 phosphorylation at TYR705 and hypoxia-induced HIF-1α accumulation in both endothelial and tumor cells

**DOI:** 10.18632/oncotarget.3648

**Published:** 2015-04-10

**Authors:** Yan Wang, Jia-Xin Li, Ying-Qing Wang, Ze-Hong Miao

**Affiliations:** ^1^ Division of Antitumor Pharmacology, State Key Laboratory of Drug Research, Shanghai Institute of *Materia Medica*, Chinese Academy of Sciences, Shanghai 201203, China; ^2^ College of Pharmacy, Nanchang University, Nanchang 330006, China

**Keywords:** tanshinone I, angiogenesis, Stat3, HIF-1α, VEGF

## Abstract

Tanshinone I (Tanshinone-1), a major active principle of *Salvia miltiorrhiza* (Danshen), has been shown to overcome tumor drug resistance and metastasis. Here we report that tanshinone-1 inhibits angiogenesis. Tanshinone-1 inhibited proliferation, migration and tube formation of vascular endothelial cells, rat aortic ring sprouting and the neovascularization of the chick chorioallantoic membrane in a concentration-dependent manner. In endothelial cells, tanshinone-1 almost completely inhibited phosphorylation of Stat3 at Tyr705 regardless of hypoxia or normoxia but only slightly decreased the hypoxia-induced HIF-1α accumulation. In tumor cells, contrastively, tanshinone-1 could not only make phosphorylation of Stat3 at Tyr705 disappear but also reduce the hypoxia-induced accumulation of HIF-1α to its baseline levels at normoxia. Consequently, VEGF secretion from tumor cells was reduced, which could potentiate the direct inhibition of tanshinone-1 on endothelial cells. Together with its overcoming tumor drug resistance and metastasis, our results reveal unique characteristics of tanshinone-1 and its improved derivatives as promising angiogenesis inhibitors.

## INTRODUCTION

Angiogenesis inhibition has become an important strategy for cancer therapy. More than 10 angiogenesis inhibitors have been used in the clinic. Most of them, either antibody agents or small-molecule drugs, target the vascular endothelial growth factor (VEGF) - VEGF receptor (VEGFR) signaling pathway. However, several issues have arisen from their applications. They offer only very limited incremental survival benefits, elicit severe toxicities especially cardiovascular toxicities and possibly stimulate treatment resistance and metastasis [1]. These issues drive explorations on targeting other drivers of angiogenesis, for instance, angiopoietin. Till now, unfortunately, the efforts have been rewarded with only limited successes. In Phase III trials in patients with ovarian cancer, the angiopoietin inhibitor trebananib increased the median overall survival by only 1 month [2, 3]. Therefore, finding new antiangiogenesis strategies becomes urgent for cancer therapy in a safe, efficacious way and ideally overcoming tumor drug resistance and/or metastasis.

**Figure 1:**
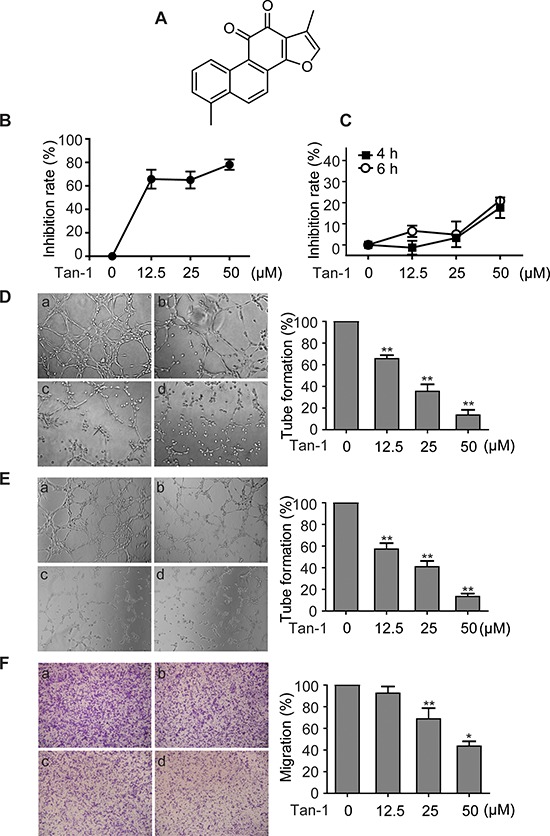
Tanshinone-1 (Tan-1) inhibits the tube formation and migration of endothelial cells **A.** Chemical structure of Tan-1. **B.** Tan-1 inhibited proliferation of endothelial cells. Human microvascular endothelial HMEC-1 cells were exposed to gradient concentrations of Tan-1 for 72 h. Proliferation inhibition of the cells was measured by the CellTiter-Glo^®^ Luminescent Cell Viability assay. **C.** Tan-1 inhibited proliferation of endothelial cells at the same cell density and exposure time to **D**, **E** or **F**. **D** to **F.** Tan-1 inhibited the tube formation of both HMEC-1 cells (**D**) and HUVEC cells (**E**) and the migration of HMEC-1 cells (**F**). Cells were treated with vehicle (a), or Tan-1 12.5 μM (b), 25 μM (c) or 50 μM (d) in the corresponding left panel for 4 h (**D** and **E**) or 6 h (**F**). Magnification: 20× (**D**) or 10× (**E** and **F**). The data from three independent experiments were expressed as mean ± SD in the corresponding right panel. *p < 0.05; **p < 0.01.

Many natural products have been reported to inhibit angiogenesis *via* various mechanisms though having scarcely been approved for cancer therapy yet due to toxicities or other reasons [4–10]. The traditional Chinese medicine *Salvia miltiorrhiza* (Danshen) is famous for its safe, effective treatment of cardiovascular diseases with a long history. Its several preparations are still widely used, especially in the treatment of angina pectoris and congestive heart failure in China [11–14]. Tanshinone I (Tanshinone-1; Figure 1A), an active principle of Danshen, shows its clinical safety based on its high content in this plant [11] and its cardiovascular activity [12]. More importantly, tanshinone-1 has been shown to kill drug-resistant tumor cells. This activity is correlated well with its reducing the active form of signal transducer and activator of transcription 3 (Stat3), phosphorylated Stat3 at Tyr705 (p-705-Stat3) [11]. Tanshinone-1 was also found to inhibit tumor metastasis by suppressing the tumor necrosis factor-α (TNF-α)-induced transcriptional activity of nuclear factor kappa B (NFκB) [15].

Here we show that tanshinone-1 inhibits angiogenesis at either hypoxia or normoxia by directly acting on both endothelial and tumor cells. Tanshinone-1 inhibited proliferation, migration and differentiation (tube formation) of endothelial cells and thus blocked angiogenesis at its initiation stage. The antiangiogenic activity was further reflected in its suppressing rat aortic ring sprouting and the neovascularization of the chick chorioallantoic membrane. The effect of tanshinone-1 on endothelial cells was correlated mainly with its reducing p-705-Stat3 at both hypoxia and normoxia though it also slightly lowered the hypoxia-induced accumulation of hypoxia inducible factor 1 alpha (HIF-1α). Moreover, this effect could be further amplified by the reduction of VEGF secretion from tumor cells subsequent to tanshinone-1-mediated decrease in p-705-Stat3 regardless of ambient oxygen conditions and hypoxia-induced HIF-1α accumulation. Together with its good safety and excellent characteristics in overcoming tumor drug resistance and metastasis, our findings could distinguish tanshinone-1 and its improved derivatives from present antiangiogenesis agents, especially those used in the clinic.

## RESULTS

### Tanshinone-1 inhibits proliferation, tube formation and migration of vascular endothelial cells

Vascular endothelial cells play critical roles in angiogenesis, especially at its initiation stage. Tanshinone-1 was shown to inhibit proliferation of human microvascular endothelial (HMEC-1) cells in a concentration-dependent manner (Figure 1B). For the 72-h treatment, tanshinone-1 had an IC_50_ value of 7.75 μM in HMEC-1 cells, which is roughly equal to its previously reported potency in tumor cells [11]. To find the proper conditions to test its effect on the tube formation and migration of vascular endothelial cells, we exposed HMEC-1 cells (2.5 × 10^4^ cells or 2 × 10^5^ cells per well) to tanshinone-1 for 4 h or 6 h. Tanshinone-1 displayed only marginal proliferation inhibition on HMEC-1 cells, and even at 50 μM, the inhibitory rate was just below 20% (Figure 1C). At the same cell density and exposure time, however, tanshinone-1 caused suppression of the tube formation of both HMEC-1 cells (Figure 1D) and human umbilical vascular endothelial (HUVEC) cells (Figure 1E) and the migration of HMEC-1 cells (Figure 1F) in a concentration-dependent manner. Proliferation could provide enough cell number of endothelial cells; migration could allow those cells to move themselves to new locations; and differentiation (here mimicked by the tube formation assay) is required for single endothelial cells to evolve finally into vascular networks. All of them are important to initiate angiogenesis. Therefore, our results suggest that tanshinone-1 could inhibit the initiation of angiogenesis at its different essentials.

### Tanshinone-1 inhibits angiogenesis both *ex vivo* and *in vivo*

**Figure 2:**
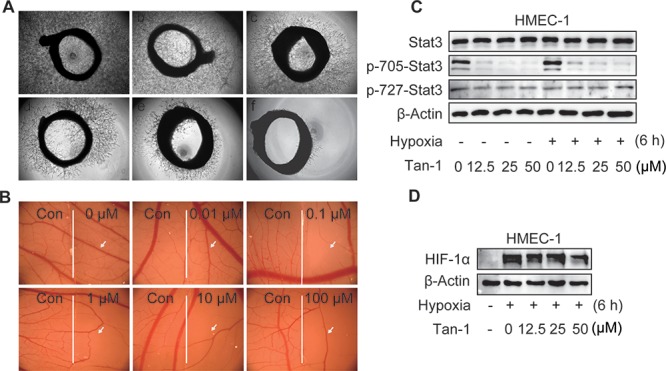
Tanshinone-1 (Tan-1) inhibits angiogenesis and reduces p-705-Stat3 and HIF-1α in HMEC-1 cells **A.** Rat aortic rings were treated with vehicle (a), Tan-1 1.25 μM (b), 2.5 μM (c), 5 μM (d), 10 μM (e), or 20 μM (f) for 7 days; magnification: 4×. **B.** Chicken eggs were treated with vehicle (the left hand side of each image) or gradient concentrations of Tan-1 (the right hand side of each image); magnification: 1.6×. **C** and **D.** Tan-1 reduced p-705-Stat3 C and HIF-1α D in HMEC-1 cells. Cells were treated with gradient concentrations of Tan-1 for 6 h at hypoxia or normoxia, and then detected by Western blotting. All experiments were independently done at least 2 times and the representative images were presented.

Assays for rat aortic ring sprouting and neovascularization of the chick chorioallantoic membrane are classical models for *ex vivo* and *in vivo* angiogenesis, respectively. Tanshinone-1 was shown to reduce both the microvessel sprouting of rat aortic rings (Figure 2A) and the microvessels of the chick chorioallantoic membrane (Figure 2B, the right panel *vs* the left one of each image) in a concentration-dependent manner. The results further indicate that tanshinone-1 could suppress not only the initiation of angiogenesis by inhibiting the functions of endothelial cells but also the final formation of blood vessels.

### Tanshinone-1 reduces p-705-Stat3 and HIF-1α in endothelial cells, which could contribute to its tube-formation inhibition

Both Stat3 and HIF-1α have been reported to be involved in angiogenesis [16, 17]. Tanshinone-1 was shown to reduce the levels of p-705-Stat3 at both normoxic and hypoxic conditions, but not to lower the levels of either the total Stat3 or phosphorylated Stat3 at Ser727 (p-727-Stat3) in HMEC-1 cells (Figure 2C). In contrast, tanshinone-1 could also decrease the levels of hypoxia-induced HIF-1α protein in the same cells in a concentration-dependent manner, but apparently weakly when compared with its effect on p-705-Stat3 (Figure 2D).

**Figure 3:**
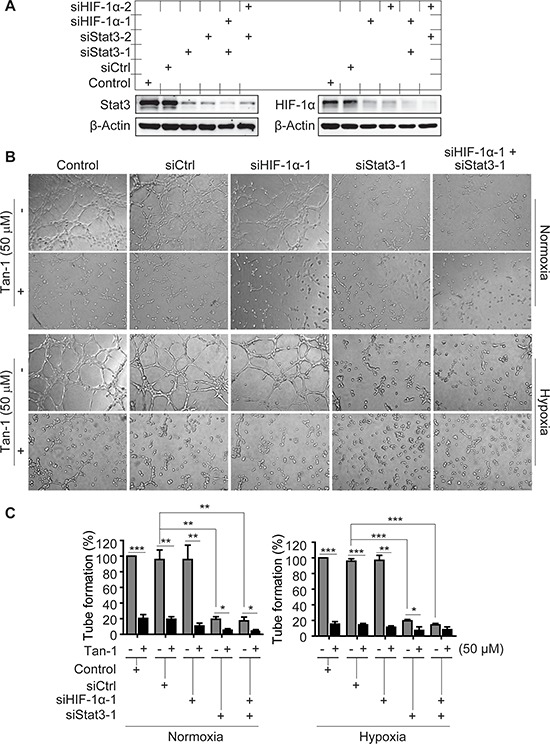
Effects of Stat3 and/or HIF-1α downregulation on the tube-formation inhibition of tanshinone-1 (Tan-1) in HMEC-1 cells **A.** HMEC-1 cells were treated with scramble siRNA (siCtrl), specific siRNA targeting human *hif-1α* cDNA (siHIF-1α-1 and -2) or *stat3* cDNA (siStat3-1 and -2) or just the transfection reagent (Control) for 48 h. And then the cells were detected by Western blotting for the levels of Stat3 and HIF-1α proteins. **B.** Following the 48-h-pretreatment of HMEC-1 cells with siCtrl, siHIF-1α-1, siStat3-1 or the transfection reagent (Control), the cells treated with or without 50 μM Tan-1 were seeded on Martigel and allowed to form tube networks for 4 h at normoxia or hypoxia; magnification: 20×. **C.** Data from three independent experiments in B were expressed as mean ± SD. *p < 0.05; **p < 0.01; and ***p < 0.001.

To detect whether the antiangiogenic activity of tanshinone-1 is linked to its effect on p-705-Stat3 or HIF-1α protein, we used specific siRNA against *hif-1α* (siHIF-1α-1 and -2 with different sequences), *stat3* (siStat3-1 and -2 with different sequences), or both to silence the corresponding genes (Figure 3A). Tanshinone-1 was shown to inhibit the tube formation of HMEC-1 cells with nearly equal potency at both normoxia and hypoxia (Figure 3B and 3C; Supplementary Figure S1A and S1B). Silencing of the *hif-1α* gene did not obviously impair the tube formation of HMEC-1 cells at either normoxia or hypoxia, but slightly potentiated the inhibitory effect of tanshinone-1 (Figure 3B and 3C; Supplementary Figure S1A and S1B). In contrast, silencing of the *stat3* gene or both *stat3* and *hif-1α* genes reduced the tube formation at both normoxia and hypoxia, which was further potentiated by the treatment with 50 μM tanshinone-1 (Figure 3B and 3C; Supplementary Figure S1A and S1B). These results demonstrate the following facts: (1) Ambient oxygen concentrations does not affect the inhibitory effect of tanshinone-1 on the tube formation of HMEC-1 cells, (2) Stat3 rather than HIF-1α stimulates the tube formation of HMEC-1 cells at both normoxia and hypoxia, and (3) Downregulation of Stat3 or HIF-1α or both could not reverse but slightly potentiates the inhibitory effect of tanshinone-1 on tube formation of endothelial cells.

### Tanshinone-1 decreases the levels of HIF-1α and p-705-Stat3 and the secretion of VEGF in tumor cells

**Figure 4:**
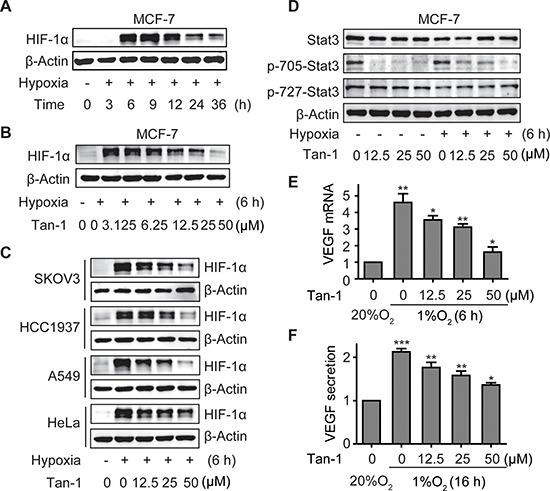
Tanshinone-1 (Tan-1) reduces the levels of HIF-1α and p-705-Stat3 and decreases the mRNA level and protein secretion of VEGF in hypoxic tumor cells **A.** MCF-7 cells were exposed to hypoxia (1% O_2_) for the indicated time, and HIF-1α levels were analyzed by Western blotting. **B** and **C.** MCF-7 B, SKOV3, HCC1937, A549 and HeLa C cells were treated with gradient concentrations of Tan-1, and immediately exposed to hypoxia for 6 h. HIF-1α levels were detected by Western blotting. **D.** Tan-1 reduced p-705-Stat3 in MCF-7 cells at either normoxia or hypoxia. The cells were treated with gradient concentrations of Tan-1, and immediately exposed to hypoxia or normoxia for 6 h. Western blotting was done to detect the levels of Stat3 and p-705-Stat3 or p-727-Stat3. **E** and **F.** Tan-1 inhibited *vegf* gene transcription (**E**) and VEGF protein secretion (**F**) in hypoxic MCF-7 cells. The cells were treated with gradient concentrations of Tan-1 at hypoxia for 6 h (*vegf* transcription) or 16 h (VEGF secretion). Then the total RNA was isolated for *vegf* mRNA analyses by Real-Time PCR, and cell supernatants were collected and measured for VEGF protein secretion by ELISA. The level of mRNA or protein at normoxia (20% O_2_) was normalized as 1. Data from three independent experiments were expressed as mean ± SD. *p < 0.05; **p < 0.01; ***p < 0.001.

VEGF, the most potent angiogenesis-stimulating factor, can be secreted from solid tumor cells in response to hypoxia stress and/or active angiogenic growth factor signaling such as the Stat3 signaling. Reduction of VEGF secretion from tumor cells might attenuate angiogenesis [18]. When MCF-7 cells were exposed to hypoxia, the level of HIF-1α first rose, peaked at 6 h – 9 h, and then declined gradually (Figure 4A). Tanshinone-1 was revealed to lower the 6-h-hypoxia-induced accumulation of HIF-1α protein in a concentration-dependent manner in different tumor cells including MCF-7, SKOV3, HCC1937, A549 and HeLa cells (Figure 4B and 4C). And its activity in reducing HIF-1α in tumor cells was more potent than in endothelial cells (Figure 2D). In contrast, tanshinone-1 elicited similar effects on Stat3 in both MCF-7 and HMEC-1 cells by decreasing p-705-Stat3 but not lowering the levels of total Stat3 or p-727-Stat3 at normoxia or hypoxia (Figure 4D). Tanshinone-1 was further shown to reduce the hypoxia-induced increase in the levels of VEGF mRNA and VEGF protein secretion in MCF-7 cells (Figure 4E and 4F).

### Tanshinone-1 decreases hypoxia-induced HIF-1α protein through the proteasome pathway in MCF-7 cells

**Figure 5:**
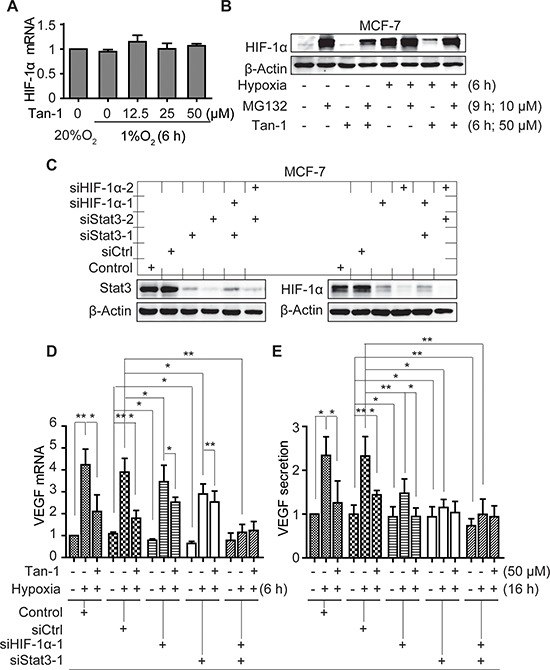
Downregulation of HIF-1α and Stat3 alleviates the reduction of VEGF mRNA and protein secretion induced by tanshinone-1 (Tan-1) in hypoxic MCF-7 cells **A.** MCF-7 cells were treated with gradient concentrations of Tan-1, and immediately exposed to hypoxia for 6 h. The levels of *hif-1α* mRNA were analyzed by Real-Time PCR. **B.** Cells were pretreated with MG-132 for 3 h, then treated with 50 μM Tan-1 and exposed to hypoxia or normoxia for another 6 h, respectively. The cells were collected for Western blotting. **C.** MCF-7 cells were pretreated with the transfection reagent (Control) or siRNA (siCtrl, siHIF-1α-1 or -2, siStat3-1 or -2 as indicated) for 48 h. Western blotting was done to detect the levels of Stat3 and HIF-1α proteins. **D** and **E.** Followed by the 48-h pretreatment with the indicated siRNA, the cells were treated with 50 μM Tan-1 and exposed to hypoxia for 6 h (**D**) or 16 h (**E**). Then Real-Time PCR and ELISA assays were done to detect the mRNA levels D and protein secretion E of VEGF, respectively. The level of mRNA or protein in the control group at normoxia was normalized as 1. Data from three independent experiments were expressed as mean ± SD. *p < 0.05; **p < 0.01; ***p < 0.001.

Hypoxia induces the accumulation of HIF-1α protein mainly by blocking its degradation *via* the ubiquitin proteasome pathway [19]. Treatments with tanshinone-1 did not change the mRNA level of *hif-1α* (Figure 5A) but decreased the protein levels of HIF-1α in MCF-7 cells (Figure 5B). MG-132, a widely used proteasome inhibitor, could enhance the levels of HIF-1α protein, especially prominently at normoxia (Figure 5B), just as previously reported [19]. Pretreatments with MG-132 effectively reversed the tanshinone-1-driven decrease of HIF-1α protein (Figure 5B). The data indicate that tanshinone-1 could decrease hypoxia-induced HIF-1α protein by impairing the proteasome-mediated degradation rather than by reducing the expression of the *hif-1α* gene.

### Both HIF-1α and Stat3 signaling contributes to the tanshinone-1-driven reduction of the mRNA levels and the protein secretion of VEGF in hypoxic MCF-7 cells

To examine whether the effect of tanshinone-1 on VEGF is correlated with its effect on p-705-Stat3 and/or HIF-1α protein in tumor cells, we used specific siRNA against *hif-1α* (siHIF-1α-1 and -2), *stat3* (siStat3-1 and -2) or both to downregulate the expression of the corresponding genes in MCF-7 cells (Figure 5C). We found that hypoxia induced the increase in the mRNA levels and the protein secretion of VEGF, which could be obviously inhibited by tanshinone-1 (Figure 4E and 4F; the groups of Control and siCtrl in Figure 5D and 5E and in Supplementary Figure S2A and S2B). Downregulation of either HIF-1α or Stat3 partially prevented the hypoxia-induced increase in the mRNA levels and the protein secretion of VEGF, which was further potentiated by adding tanshinone-1. In contrast, simultaneous downregulation of both HIF-1α and Stat3 completely prevented the hypoxia-induced VEGF increase. Moreover, the addition of tanshinone-1 did not further lower the levels of the VEGF mRNA or the secreted VEGF protein (Figure 5D and 5E; Supplementary Figure S2A and S2B). These results indicate that both HIF-1α and Stat3 signaling contributes to the effect of tanshinone-1 on the mRNA levels and the protein secretion of VEGF in hypoxic MCF-7 cells.

## DISCUSSION

**Figure 6:**
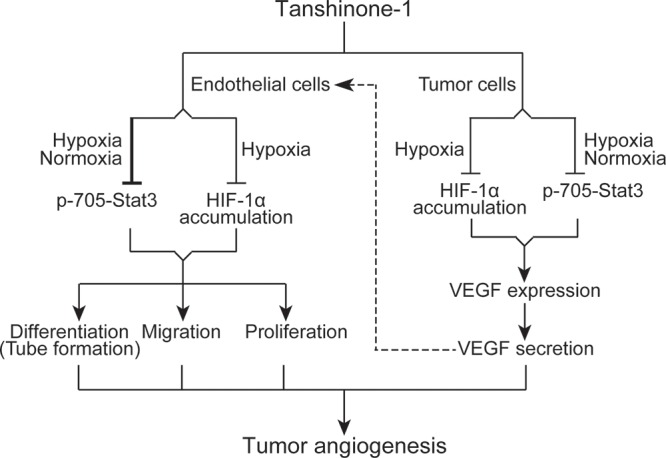
Schematic presentation of possible mechanisms by which tanshinone-1 inhibits tumor angiogenesis

In this study, we demonstrate that tanshinone-1 inhibits tumor angiogenesis in both direct and indirect ways. Tanshinone-1 directly suppresses proliferation, migration and tube formation (differentiation) of endothelial cells, which could be amplified indirectly by its reducing the secretion of VEGF from tumor cells. Tanshinone-1 attenuates the sprouting of rat aortic rings and the neovascularization of the chick chorioallantoic membrane. The data suggest that tanshinone-1 could not only block the endothelial-cell-mediated initiation stage but also impair the whole process of angiogenesis. In solid tumors, besides, tanshinone-1 reduces VEGF paracrine from tumor cells, which might prevent the stimulation of VEGF on endothelial cells (Figure 6), further strengthening its antiangiogenesis activity.

Both Stat3 and HIF-1α have been revealed to drive tumor angiogenesis, metastasis and drug resistance [20–22]. We previously reported that reduction of p-705-Stat3 or HIF-1α accumulation could overcome drug resistance [6, 11]. Stat3 has also been shown to form transcription complexes with NFκB, which synergistically drives the expression of the metastasis-driving factors [23]. So tanshinone-1-induced reduction of p-705-Stat3 might also contribute to its reported antimetastasis activity [15]. In this study, we show that tanshinone-1 decreases p-705-Stat3 regardless of the ambient oxygen conditions in both endothelial and tumor cells. Moreover, downregulation of Stat3 abrogates tube formation of HMEC-1 cells and VEGF secretion from MCF-7 cells, which is further potentiated by tanshinone-1. These data suggest a possibility that tanshinone-1 could inhibit angiogenesis through the whole solid tumor, including its hypoxic and non-hypoxic regions.

Stat3 has been demonstrated to directly promote the expression of HIF-1α [22, 24]. However, the tanshinone- 1-driven reduction of hypoxia-induced HIF-1α accumulation could not be correlated with its effect on Stat3 in either endothelial or tumor cells. In the identically-treated endothelial cells, tanshinone-1 reduces p-705-Stat3 but only slightly lowers HIF-1α accumulation. Abrogating the expression of HIF-1α does not block the tube formation or obviously change the effect of tanshinone-1 on the tube formation either. Similarly in tumor cells, tanshinone-1 does not alter the mRNA levels of HIF-1α in tumor cells whereas the proteosome inhibitor MG-132 prevents the reduction of HIF-1α accumulation induced by tanshinone-1. These data indicate that tanshinone-1 inhibits Stat3 and HIF-1α possibly by independent mechanisms. In contrast, these independent changes in Stat3 and HIF-1α induced by tanshinone-1 synergistically inhibit the tube formation of endothelial cells and the VEGF secretion from tumor cells.

Together, as an angiogenesis inhibitor, tanshinone-1 displays unique characteristics as follows. 1) Tanshinone-1 reduces p-705-Stat3 possibly by certain unknown approach(es) different from those used by the known Stat3 inhibitors [25] as we reported previously [11]. 2) Tanshinone-1 lowers the accumulation of HIF-1α, a principal regulator of hypoxia, in tumor cells, which could alleviate the ill biological effects of hypoxia. On the other hand, its direct tumor cell killing might ease the demand for oxygen in the tumor. Both help to improve tumor treatment [1]. 3) Tanshinone-1 has been shown to overcome tumor drug resistance [11] and inhibit metastasis [15], which might help to prevent and/or delay treatment resistance and metastasis resulting from antiangiogenic therapy [1]. 4) Importantly, tanshinone-1 is one of major active ingredients of Danshen, and Danshen has been safely used in the clinic for a very long time. Moreover, tanshinone-1 itself possesses therapeutic effects on cardiovascular diseases [12]. Thus, tanshinone-1 might have good safety and especially avoid causing severe cardiovascular toxicities that frequently occur in the use of most antiangiogenic agents at present. These characteristics could be reasonable to make tanshinone-1 distinct from other angiogenesis inhibitors. However, its drug development as an angiogenesis inhibitor requires further demonstrating its molecular mechanisms and improving its biological activity and physicochemical properties, both of which are underway.

## MATERIALS AND METHODS

### Cell culture

Human microvascular endothelial HMEC-1 cells were obtained from the French National Institute of Health and Medical Research (INSERM, FR), and cultured with complete MCDB131 medium (Invitrogen, Carlsbad, CA, USA). HUVEC cells were purchased from AllCells (Shanghai, China). Human breast cancer MCF-7 and HCC1937, lung cancer A549, cervical cancer HeLa and ovarian cancer SKOV3 cells were all purchased from the American Type Culture Collection (ATCC, Manassas, VA, USA). Cells were normally cultured in the ATCC-specified medium in humidified atmosphere containing 5% CO_2_ at 37°. Hypoxia treatments were performed by putting the tested cells in a humidified atmosphere containing 5% CO_2_ at 1% oxygen partial pressure (Ruskin Invivo 400 system; Ruskin Technology Ltd, Bridgend, UK).

### Drugs

Tanshinone-1 and MG-132 were purchased from Selleck (Houston, TX, USA). Antibodies against p-705-Stat3, p-727-Stat3 and Stat3 were purchased from Cell Signaling Technology (Beverly, MA, USA). The antibody against HIF-1α and the growth factor reduced matrigel (BD354230) were obtained from BD Biosciences (Becton Dickinson Labware, MA). The antibody against *β*-Actin was purchased from Santa Cruz Biotechnology (Santa Cruz, CA, USA).

### Proliferation inhibition assays

Proliferation-inhibitory effects of tanshinone-1 on HMEC-1 cells were assessed by the CellTiter-Glo^®^ Luminescent Cell Viability Assay as reported previously [26]. HMEC-1 cells (8 × 10^3^ cells per well for a 72-h assay) were seeded to 96-well plates for attachment overnight (for the 72-h assay). Then the cells were treated with gradient concentrations of tanshinone-1 for 72 h. On the other hand, to find the proper experimental conditions for the following tube formation and cell migration assays, HMEC-1 cells (2 × 10^5^ cells per well for a 6-h assay and 2.5 × 10^4^ cells per well for a 4-h assay) were seeded to 96-well plates and then treated with gradient concentrations of tanshinone-1 immediately for 4 h or 6 h. The cell viability was examined by using the CellTiter-Glo^®^ Luminescent Cell Viability Assay kit (Promega, Madison, WI). Luminescence was recorded with an EnVision Multilabel Reader (PerkinElmer, Waltham, MA).

### Tube formation assays

Chilled liquid Matrigel was dispensed onto 96-well plates (50 μL per well) and allowed to solidify (37°, 1 h) [27]. Then HMEC-1 cells or HUVEC cells (2.5 × 10^4^ cells per well) were seeded onto the gel and cultured in the medium containing tanshinone-1 at 37° for 4 h. The enclosed networks of complete tubes from three randomly chosen fields were counted and photographed under a microscope (Olympus, Tokyo, Japan).

### Cell migration assays

The migration assay was done in a transwell Boyden chamber (Costar, MA, USA). HMEC-1 cells (2 × 10^5^ cells per well) in the serum-free MCDB131 medium containing tanshinone-1 (0, 12.5, 25 or 50 μM) were added to the upper compartment of the chamber. The lower compartment contained MCDB131 medium supplemented with 20% fetal bovine serum (FBS) and tanshinone-1. After 6-h incubation at 37°, the cells still staying on the upper face of the transwell membrane were removed with a cotton swab. The migrated cells on the lower side of the transwell membrane were fixed with 90% ethanol and then stained with 0.1% crystal violet in 0.1 M borate and 2% ethanol (pH 9.0). After being photographed, the stained cells were subsequently extracted with 10% acetic acid. The absorbance values were determined at 600 nm with the SpectraMAX190 of Molecular Devices (Sunnyvale, CA).

### Aortic ring sprouting assays

Aortic rings from 6-week-old Sprague Dawley rats [28, 29] were dissected into 1-mm-long rings. The rings were embedded with 70-μL Matrigel, allowed to solidify (37°, 1 h) and then incubated with tanshinone-1 in the serum-free M199 medium for 7 days. The aortic ring sprouts were photographed with a microscope (Olympus, Tokyo, Japan). All animal procedures and experiments complied with the ethical Guidelines for the care and use of animals and were approved by the Institute Animal Review Boards of the Shanghai Institute of *Materia Medica* at the Chinese Academy of Sciences.

### Chick chorioallantoic membrane assays

Fertilized chicken eggs were incubated in an egg incubator (Lyon, CA) at 37° and 50% humidity for 7 days. Then the alive embryos were chosen and the chorioallantoic membranes were treated with different concentrations of tanshinone-1 (0, 0.01, 0.1, 1, 10, 100 μM) dried on coverslips for 48 h, as described previously [30]. The vascular images were captured by stereomicroscopic photography (MSS, Leica, Swizerland). Antiangiogenic effect was detected by counting the number of blood vessel branch points under the coverslips with tanshinone-1 (the right side of each image) or just with the vehicle (the left side of each image). At least 10 viable embryos were enrolled for each concentration group.

### Western blotting analyses

The tested endothelial cells and tumor cells (1.5 × 10^5^ cells per well) were exposed to different concentrations of tanshinone-1 at hypoxic or normoxic conditions for the indicated time. Then the cellular levels of proteins were determined as previously described [31–34].

### Real-time PCR

Total RNA of cells was extracted using the TRIzol (Invitrogen, Carlsbad, CA, USA). One microgram of total RNA was reverse-transcribed using a RT reagent kit (TaKaRa, Tokyo, Japan). The cDNA was amplified with a 7500 Fast Real-time PCR System (Applied Biosystem, Grand Island, NY, USA). The primer sequences were as follows: 5′-GCTGTCTTGGGTGCATTGGA-3′ (forward) and 5′-ATGATTCTGCCCTCCTCCTTCT-3′ (reverse) for *vegf*; 5′-CCAGCAGACTCA AATACAAGAACC-3′ (forward) and 5′-TGTATGTGGGTAGGAGATGGAGAT-3′ (reverse) for *hif-1α*; 5′-GGATGCAGAAGGAGATCACTG-3′ (forward); and 5′-CGA TCCACACGGAGTACTTG-3′ (reverse) for *β-actin*. The relative quantification of mRNA levels was automatically done with the software of the PCR system.

### ELISA kinase assays

MCF-7 cells were seeded in a 6-well plate at a density of 1.5 × 10^5^ cells per well. After attachment, the medium was replaced with fresh medium supplemented with 1% FBS, and the cells were treated with tanshinone-1 at hypoxia or normoxia for 16 h. Cell supernatants were collected, and pellets were harvested by trypsinization to calculate the cell number. The amount of VEGF in the supernatant was determined with a human VEGF-ELISA kit (Multi Sciences, Shanghai, China) according to the manufacturer's instructions. VEGF was expressed as pictograms of VEGF protein per milliliter of medium and per 10^5^ cells.

### Small interfering RNA (siRNA)

Cells were transfected with 100 nM siRNA using RNAi Max (Invitrogen, Carlsbad, CA, USA) according to the manufacturer's instructions. The sequences of siRNA were as follows: 5′-GUUUGGAAAUAAU GGUGAATT-3′ (siStat3-1) and 5′-GAGUUGAAUUAUCAGCUUATT-3′ (siStat3-2) for *stat3*; 5′-CACCAUGAUAUGUUUACUATT-3′ (siHIF-1α-1) and 5′-AGGACAAGUCACAACAGGAUU-3′ (siHIF-1α-2) for *hif-1α*; and 5′-UUCUCCGAACGUGUC ACGUTT-3′ as negative control (siCtrl). These siRNA sequences were synthesized by Shanghai GenePharma Co. Ltd (Shanghai, China).

## SUPPLEMENTARY FIGURES


