# Supplementary material for: Tanshinone I inhibits tumor angiogenesis by reducing STAT3 phosphorylation at TYR705 and hypoxia-induced HIF-1α accumulation in both endothelial and tumor cells
Source: Oncotarget. 2015 Apr 10;6(18):16031–42. doi: 10.18632/oncotarget.3648 (PMC4599254; doi:10.18632/oncotarget.3648)
Supplement: Supplementary file 1 [file oncotarget-06-16031-s001.pdf]

SUPPLEMENTARY FIGURES

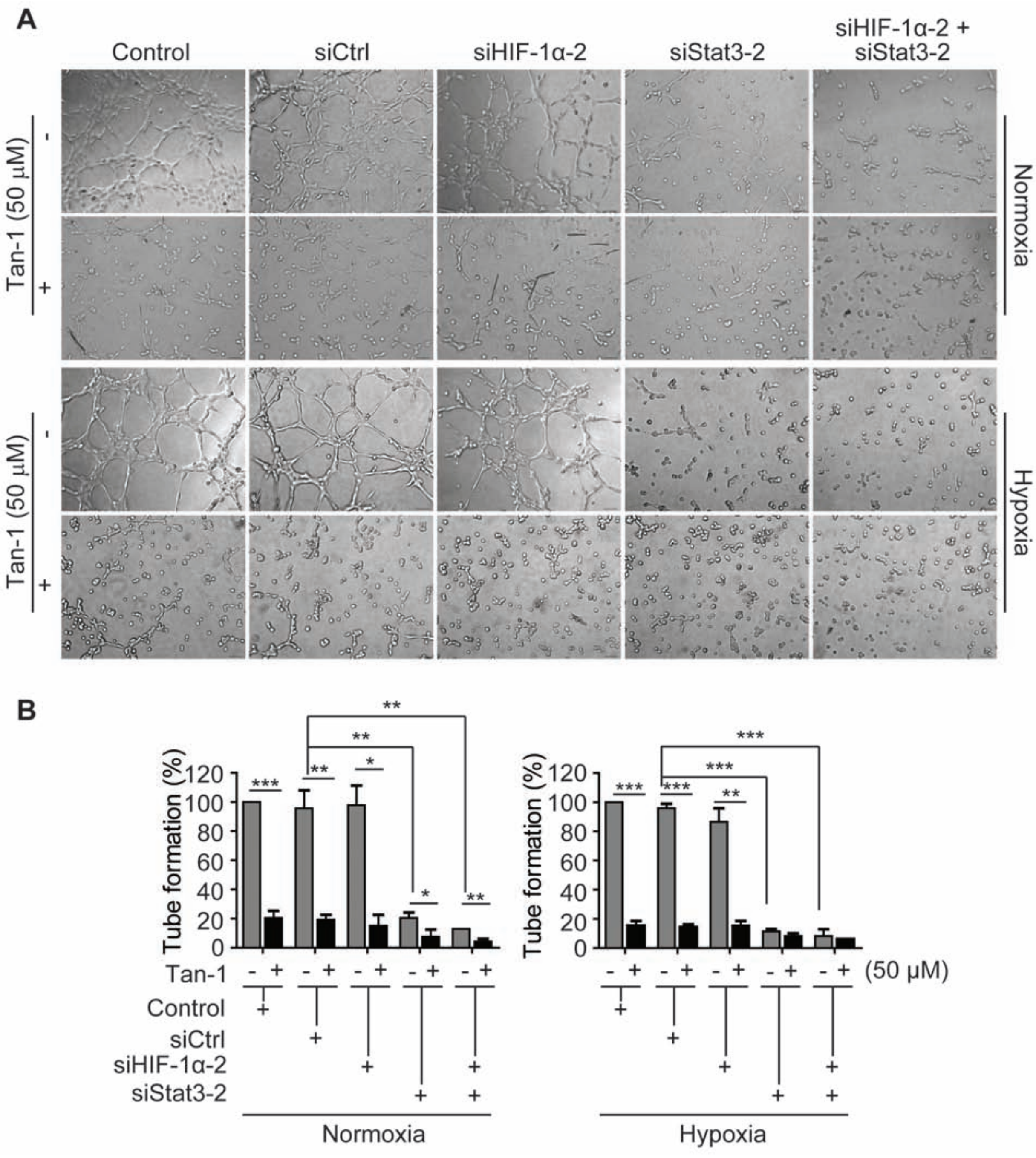

Supplementary Figure S1: Effects of Stat3 and/or HIF-1α downregulation on the tube-formation inhibition of tanshinone-1 in HMEC-1 cells.

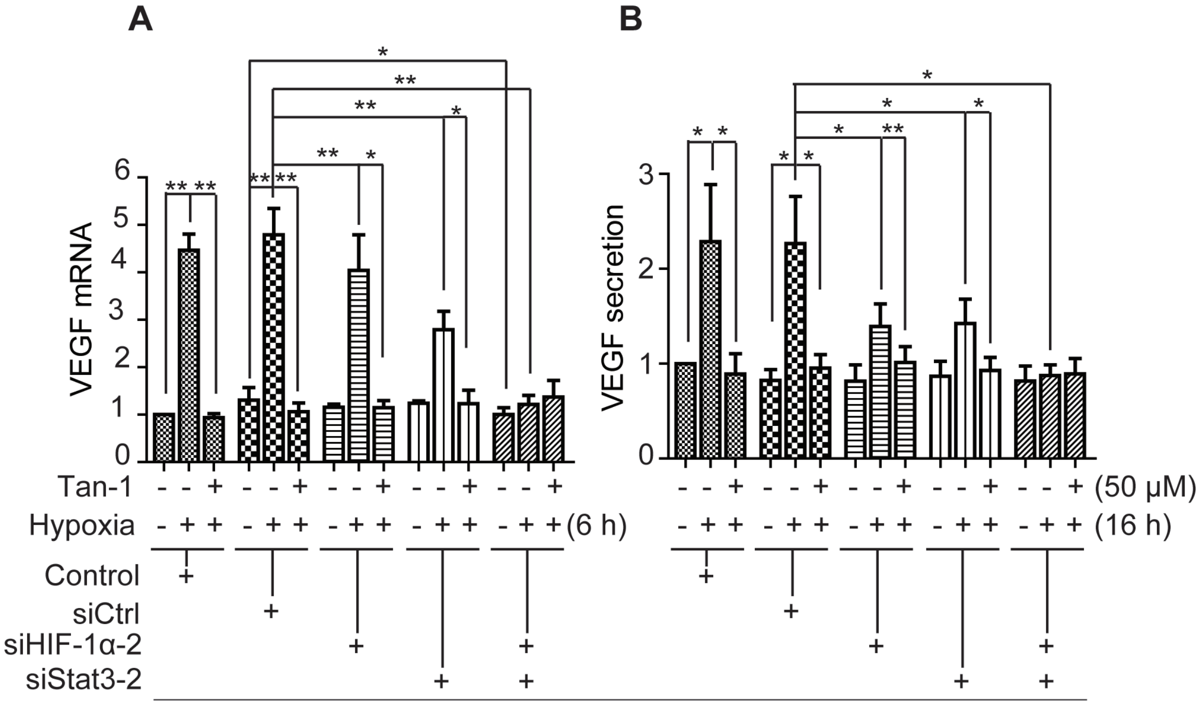

Supplementary Figure S2: Downregulation of HIF-1α and Stat3 alleviates the reduction of VEGF mRNA and protein secretion mediated by tanshinone-1 (Tan-1) in hypoxic MCF-7 cells.
